# Expression of placental growth factor mRNA in preeclampsia

**Published:** 2017-03

**Authors:** Pooneh Nikuei, Minoo Rajaei, Kianoosh Malekzadeh, Azim Nejatizadeh, Fatemeh Mohseni, Fatemeh Poordarvishi, Nasrin ghashghaeezadeh, Mehrdad Mohtarami

**Affiliations:** 1 *Molecular Medicine Research Center, Hormozgan Health Institute, Hormozgan University of Medical Sciences, Bandar Abbas, Iran.*; 2 *Fertility and Infertility Research Center, Hormozgan University of Medical Sciences, Bandar Abbas, Iran.*; 3 *Department of Medical Genetics; Faculty of Medicine; Hormozgan University of Medical Sciences, Bandar Abbas, Iran.*; 4 *Biology Department, Long Island University, Post Campus, New York, USA.*

**Keywords:** Preeclampsia, Expression, Placental growth factor, Endothelial dysfunction

## Abstract

**Background:**

Preeclampsia (PE) is a serious complication of pregnancy with hallmarks of incomplete placentation, placental ischemia and endothelial dysfunction. Imbalance between vascular endothelial growth factor (VEGF), placenta growth factor (PlGF) and their receptors play important role in pathophysiology of PE.

**Objective:**

This study was aimed to asses PlGF mRNA expression in placenta of women affected with PE.

**Material and Methods:**

In this cross-sectional study, expression of PlGF mRNA was evaluated in 26 mild PE cases, 15 severe preeclamptic women and 20 normotensive controls. Patients were sub-classified as early onset PE (9) and late onset (32). After RNA extraction, PlGF expression was quantified with qRT-PCR.

**Results:**

The results of PlGF mRNA expression between mild-severe, and early-late onset PE patients showed no statistically significant difference compared with the control group (p=0.661, p=0.205 respectively).

**Conclusion:**

Despite we found no distinct differential expression of PlGF mRNA in placental tissue of PE patients compared with control women, but according to decreased level of this angiogenic factor in PE even before clinical onset of the disease, determining molecular mechanisms related to reduced secretion of PlGF into the maternal circulation may be useful for future therapeutics.

## Introduction

Preeclampsia (PE) is a serious complication of pregnancy and main cause of maternal death which causes almost 15-20% of pregnancy-related mortalities (1). PE is characterized by hypertension and proteinuria after 20^th^ wk of gestation; a multisystem disease affecting liver, kidneys, hematological and nervous system causing cerebral edema, seizures, and even maternal death (2). Currently, there is no definite treatment for PE and the only definite management is termination of pregnancy, delivery of the placenta and fetus which enhances risk of disability and death for baby especially in severe and early onset cases (3, 4). Risk factors and past obstetrics history alone cannot predict PE, and for this reason, identification of high risk women for developing PE is important (5). Incomplete placentation, placental ischemia, and endothelial dysfunction are recognized as the main pathogenesis and hallmarks of PE (6). Angiogenesis which is essential for trophoblastic invasion and uteroplacental vascularization is disrupted in PE patients but molecular mechanisms are not completely understood (7, 8). For effective angiogenesis, vasculogenesis and adequate placental development in pregnancy, a balance between vascular endothelial growth factor (VEGF), placenta growth factor (PlGF) and their receptors are crucial (9, 10). VEGF-A acts through enhancing angiogenesis as an important growth factor for endothelium (8). 

In addition to VEGF-A, PlGF induces angiogenesis and affects proliferation and migration of endothelial cells (11). PlGF belongs to VEGF family and is a pro-angiogenic factor which is closely related to VEGF-A (12). Despite existence of some angiogenic growth factors in placenta, PlGF expression has a great importance (13). In human PlGF gene is mapped to chromosome 14q24 (14). PlGF protein which shows a main homology with VEGF, is predominantly expressed in trophoblast cells of placenta and its aberrant expression could lead to insufficiency in placental vasculature (11).

VEGFR-1 (also known as Flt-1) and VEGFR-2, (also known as KDR) are two principal receptors for VEGF-A and PlGF. VEGF-A acts through both FLT1 and KDR while PlGF acts only through FLT1, and not through KDR. VEGF-A controls angiogenesis up to 25 weeks of gestation and from that time to end of pregnancy angiogenesis is regulated by PlGF (12). It is likely that PlGF displaces VEGF from VEGFR1 and pushes VEGF for binding to VEGFR2 which has kinase activity ten-fold more than VEGF1 to enhance angiogenesis (15). Also, it is proposed that PlGF could increase maturation of uterine natural killer cells for enhancing trophoblastic invasion (16).

Actually, pro-angiogenic proteins such as VEGF and PlGF, which are associated with adequate vascular endothelial homeostasis, are antagonized by sFlt-1 (17). Animal studies showed that increased levels of anti-angiogenic proteins like sFlt-1 cause symptoms including proteinuria, hypertension, hematologic abnormalities, cerebral edema, and fetal growth restriction which are observed in human preeclampsia (18-20). Due to shallow invasion and abnormal placentation which leads to hypoxia, antiangiogenic factors like sFlt-1 are released from placenta and neutralize VEGF and PlGF mediated signaling leading to endothelial dysfunction in PE patients (15). Mechanisms which enhance angiogenesis and vascular remodeling in uteroplacental unit are not completely understood (8). Many molecular pathways are considered to be involved in placentation defects like PE, but among them VEGF family mediated angiogenic pathway is known to play a key role (12). PlGF produced by placenta is released in maternal circulation (8). In normal pregnant women, PlGF level increases remarkably from first trimester and reaches to peak level at 28-30 wk of gestation and then decreases continuously from late second trimester to term. Most of studies about PlGF in PE discussed circulating levels in serum (21, 22).

To the best of our knowledge, most studies related to mRNA expression of VEGF family in PE have concentrated on VEGFA and few studies are about PlGF mRNA expression in PE. Therefore, this study was aimed to evaluate the PlGF expression in placenta tissue of women affected to PE.

## Materials and methods

Participants of this cross-sectional study were 41 women affected with PE and 20 term healthy pregnant women from two women hospitals in south of Iran. The sample size was calculated using G*Power software. The study was conducted from September 2014 to November 2015. In PE women, 26 had mild PE and severe form of disease was seen in 15 cases. These women also were classified as early onset PE (n=9) and late onset (n=32). 

Women affected with PE (18-40 yr old) were included as case group and normal term pregnant women at the same gestational age were participated as control group. Women with diabetes mellitus, renal disease, collagen vascular diseases, chronic or gestational hypertension were excluded from this study. PE was defined as gestational hypertension (systolic pressure >140 mmHg or diastolic blood pressure >90 mmHg on two or more occasions after gestational wk 20) with proteinuria (>0.3 g/day). 

If more than one of the following criteria were present, PE was defined severe: (i) severe gestational hypertension means systolic pressure >160 mm Hg or diastolic blood pressure >110 mm Hg on two or more occasions after gestational wk 20, (ii) severe proteinuria means protein ≥5 gr in a 24 hr urine specimen, (iii) oliguria, (iv) cerebral or visual disturbances, (v) pulmonary edema or cyanosis, (vi) epigastric or right upper-quadrant pain, (vii) impaired liver function, (viii) thrombocytopenia or (ix) fetal growth restriction (23). PE was also classified to early-onset (<34 wk of gestation) and late-onset (≥34 wk) (24).

Placental tissues biopsies (2 cm) were collected immediately after delivery near umbilical cord insertion and were put in RNAlater (Qiagen, Germany) and stored at -80°C until used. Total RNA was extracted from 100 mg of placental tissue biopsies using TRIZOL Isolation Reagent (Sigma-Aldrich.INC) according to the manufacturer’s instructions. RNA contaminants were removed by treatment with the RNase-free DNase-I (Thermo Scientific, USA). First RNA was treated with 1µl DNase-I in 37^o^C for 30 min and then after adding 1µl 50mM EDTA, it was incubated in 65^o^C for 10 min to inactivate DNase. RNA quality and quantity were evaluated by agarose gel electrophoresis and NanoDrop ND-1000 spectrophotometer (Thermo Scientific, USA). 

**Quantitative RT-PCR**

For each sample, 2µg of RNA was reverse transcribed to cDNA using Revert Aid TM First Strand cDNA Synthesis Kit (Fermentas, Canada) using random hexamer primer and according to the manufacturer’s protocol. Quantitative RT-PCR was done by real-time PCR machine (Corbett Rotor-Gene 6000 Australia). Primers displayed in table I and Syber Green-Master Mix (Takara syber premix ex taq, Japan) were used for qRT-PCR. β-Actin was used as endogenous control for normalization of the raw data. Reactions were carried out in 20 µL of mixture with 2 µL cDNA, master mix 2X, ROX dye 50X and 10 pmol of each primer pairs for PlGF and β-actin. The thermal cycling conditions were initial denaturation at 95^o^C for 30 sec with 40 cycles of denaturation at 94^o^C for 5 sec, annealing at 59^o^C for 15 sec, extension at 72^o^C for 30 sec. 2^-∆∆ct^ method was used for Analysis of Relative Gene Expression.

**Ethical consideration**

Informed written consent was obtained from the enrolled subjects for placental tissue collection under the protocols approved by the Ethical Committee of Hormozgan University of Medical Sciences, Hormozgan, Iran. 

**Statistical analysis**

All data analyses were performed using Graphpad prism software (version 5.0) (GraphPad Software Inc., San Diego, CA). Based on Kolmogorov-Smirnov test, data did not have normal distribution and were compared using Kruskal-Wallis test. P<0.05 was considered significant. 

## Results

Mean maternal age in PE patients was 27.83±5.89 year and 26.85±4.84 yr in controls which the difference between two groups was not statistically significant (p=0.816) and shows a matching between case and control group. Also mean body mass index (BMI) was 23.82±3.88 in case group and 23.31±3.49 in control group with no significant difference (p=0.385). Placental weight was significantly reduced in PE group (p˂0.001). 14.6% of women in PE group had history of PE in their previous pregnancies. Clinical characteristics are represented in table II.

**Figure 1:**
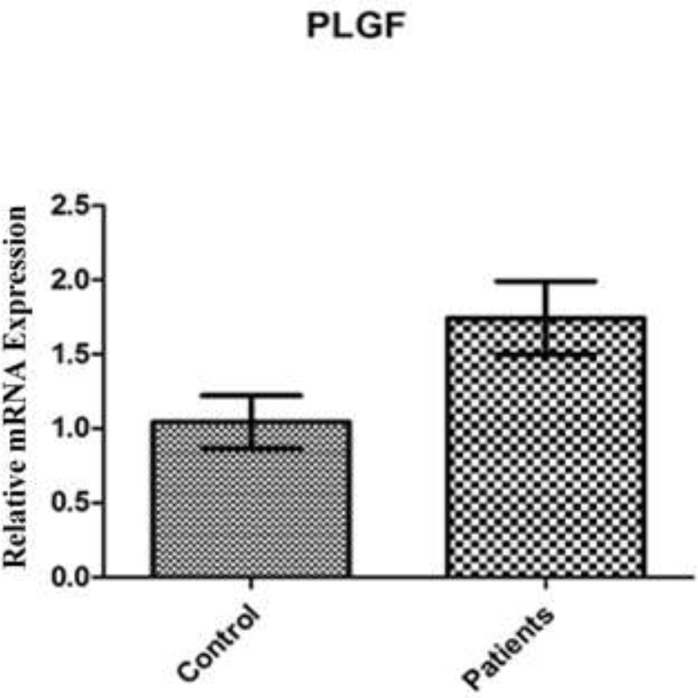
PlGF mRNA expression among patients with PE in comparison with control group. an up-regulation in PlGF mRNA was observed in patients compared to normal women which was not significant. Data are presented as means ± S.E.M and analyzed using Mann–Whitney U-test. No significant statistical difference was observed between study groups

**Figure 2:**
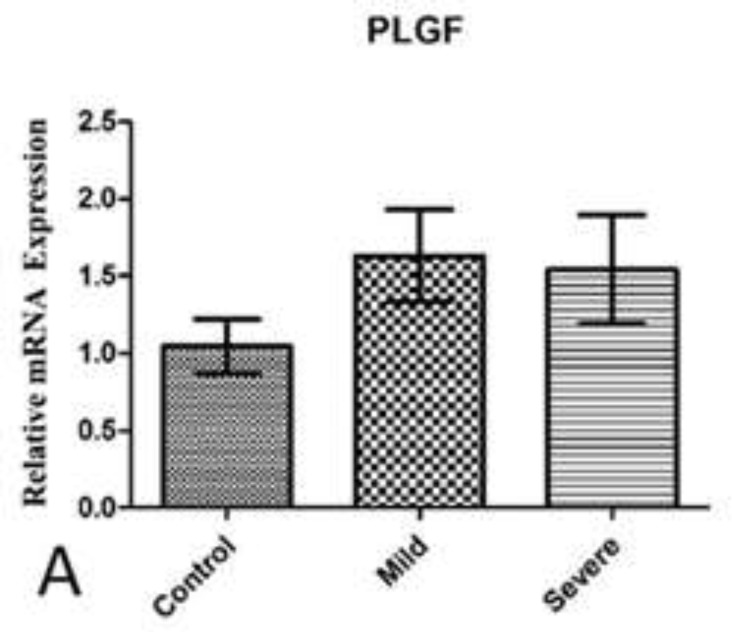
PlGF mRNA expression among Patients with mild and severe PE in comparison with control group(A). Data are presented as means ± S.E.M and analyzed using Kruskal Wallis test. No significant statistical difference was observed between study groups

**Figure 3:**
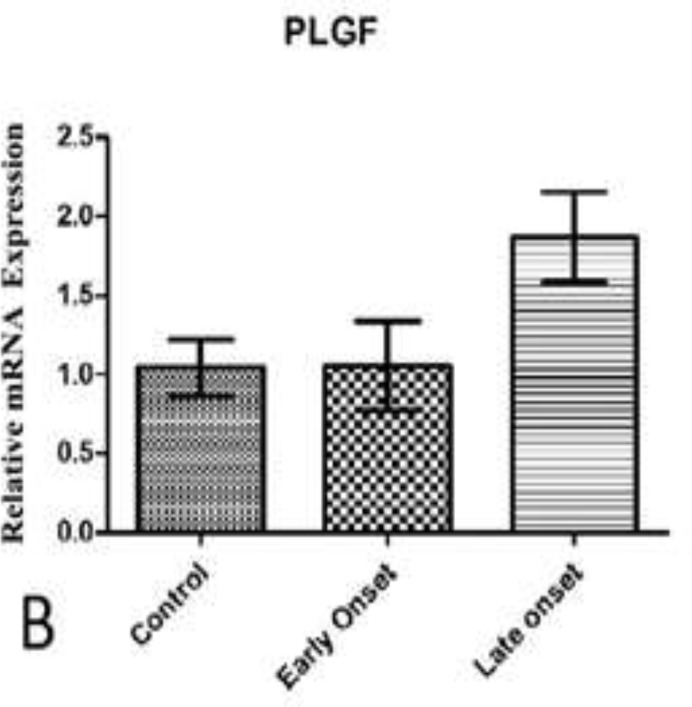
PlGF mRNA expression among Patients with early and late onset PE in comparison with control group(B). Data are presented as means ± S.E.M and analyzed using Kruskal Wallis test. No significant statistical difference was observed between study groups

Although an up-regulation in PlGF mRNA was observed in patients compared with the control women, it was not statistically significant (p=0.227). The results of PlGF mRNA expression between mild, severe and control group showed no statistically significant difference (p=0.661). Patients affected with mild and severe PE showed approximately the same expression in PlGF mRNA level. Also, mRNA expression for PlGF between early onset, late onset and control group showed no statistically significant difference (p=0.205), although an upregulation was observed in late onset patients compared with control women which was not significant. Results are shown in figures 1-3.

**Table I:** Characteristics of primers used in quantitative real-time PCR

**Gene**	**Sequence of primers 5'→ 3'**	**Size (bp)**	**An. Temp. (˚C)**
PlGF	F GGCTGTTCCCTTGCTTCCT	110	59
	R TACCACTTCCACCTCTGACGA		
β-actin	F GCCTTTGCCGATCCGC	90	58
	R GCCGTAGCCGTTGTCG		

An. Temp: annealing temperature

F: forward primer

R: reverse primer

PlGF: Placental growth factor

β-actin: βeta Actin

PCR: polymerase chain reaction

**Table II:** Clinical characteristics of PE patients and control group

**Parameter**			**PE (41)**	**Controls (20)**	**P-value**
Maternal age (yr)			27.83 ± 5.89	26.85 ± 4.84	0.816
Placental weight (gr)			427 ± 85.04	609 ± 49.41	˂0.001
BMI before pregnancy (kg/m^2^)			23.82 ± 3.88	23.31 ±3.49	0.385
Previous PE					
	yes		6 (14.6%)	0 (0.0%)	-----
	no		35 (85.4%)	20 (100.0%)	
Severity					
	Mild		26 (63.5%)	0	-----
	Severe		15 (36.5%)		
Onset Time					
	Early		9 (22%)	0	-----
	Late		32 (78%)		

Results are represented as either mean ± SD or N (%). P˂0.05 is considered to be significant.

BMI: Body Mass Index

PE: preeclampsia

## Discussion

PlGF is highly expressed in placenta in all gestational stages. It is suggested to control growth and differentiation of trophoblastic cells and proposed to have role in trophoblast invasion into the maternal decidua (14). In PE patients, the circulating levels of PlGF decreases even several weeks prior to clinical symptoms of PE (25). Polliotti and coworkers reported decreased levels of PlGF in women affected to severe early onset PE (26). Schmidt and coworkers studied about angiogenic and antiangiogenic factors in PE and reported significantly lower levels of PlGF than normotensive pregnant women (27). 

Shibata and coworkers reported lower levels of PlGF in serum samples of women affected to PE compared with normal controls (28). Meanwhile most studies related to sFlt-1 level in pregnancy shows elevated levels in peripheral blood of women with PE (18, 29). Decreased circulating PlGF level which is observed in PE patients could be explained by antagonising by sFlt-1 as a potent anti-angiogenic factor. 

Our results showed no significant difference in mRNA expression in PE patients compared with normal controls. Only a few studies reported mRNA alterations in patients affected to PE (12, 29, 32). Our results are in accordance with the findings of previous studies which reported no significant difference between the level of PlGF mRNA expression in PE women compared to normal controls (8, 30, 31). Tsatsaris and coworkers studied about dysregulation in the VEGF family in PE and reported no difference in PlGF mRNAs expression among the early onset severe PE patients and healthy controls (8). 

TOFT and colleagues worked on expression of genes which regulate angiogenesis in PE including PlGF in placentas from preeclamptic women by whole-genome microarray and reported no difference between this group and controls (30). Also Pramatirta and coworkers reported no difference between expression of cell-free mRNA of PlGF between serum of severe preeclamptic women and those with normal pregnancy (31). 

Our findings disagree with some studies reporting decreased expression of PlGF mRNA in PE patients (12, 29, 32). Andraweera and coworkers; reported reduced expression of PlGF mRNA in placental of PE patients (12). Maebayashi and colleagues reported a significant decrease in PlGF mRNA in placenta of PE patients compared to the controls (29). Purwosunu and coworkers studied about the expression of genes related to angiogenesis in blood of preeclamptic women and the results of their study showed reduced expression of PlGF mRNA (32).

Hongling and colleagues reported lower levels of PlGF transcription in PE placenta compared to normal controls (33). Also, Shibata and coworkers assessed PlGF concentration in placental villous homogenates by ELISA and reported it in patients about half that of the controls (28). Semczuk and coworkers studied about expression of genes coding for proangiogenic factors in women affected to PE and reported higher levels of PlGF transcription in PE patients (34). This study is the only one which reports up-regulation of PlGF mRNA in placenta of PE patients.

Although most studies about circulating PlGF in PE showed decreased levels even several weeks before the onset of PE, but there is an inconsistency about PlGF mRNA expression in PE. PlGF expression could be regulated at a posttranscriptional level, which explains the inconsistent results between its transcripts and protein levels (14). For better understanding of molecular mechanism of this disease, more researches with larger sample sizes and different ethnicities are needed to elucidate exact role of PlGF mRNA in PE.

## Conclusion

In conclusion, although we did not find any significant difference in placental PlGF mRNA expression in PE patients compared to control women, studies about molecular mechanisms responsible for regulating expression of PlGF in trophoblastic cells could lead to new steps towards molecular aspects of PE. 

## Note

***This article extracted from Doctoral dissertation. (Pooneh Nikuei)***
